# Intermittent fasting for obesity and related disorders: unveiling myths, facts, and presumptions

**DOI:** 10.20945/2359-3997000000322

**Published:** 2021-01-14

**Authors:** Bruno Halpern, Thiago Bosco Mendes

**Affiliations:** 1 Hospital 9 de Julho São Paulo SP Brasil Grupo de Controle do Peso, Hospital 9 de Julho, São Paulo, SP, Brasil.; 2 Universidade do Estado de São Paulo Departamento de Medicina Interna Botucatu SP Brasil Departamento de Medicina Interna, Universidade do Estado de São Paulo (Unesp), Botucatu, SP, Brasil.

**Keywords:** Obesity, intermittent fasting, type 2 diabetes, weight loss, diets, insulin resistance, circadian rhythm

## Abstract

Intermittent fasting (IF) is an increasingly popular method of weight loss, as an alternative to daily caloric restriction (DCR). Several forms of IF exist, such as alternate-day fasting or time-restricted feeding regimens. Some of its proponents claim several health benefits unrelated to caloric restriction or weight loss, which rely mainly on animal models. Although several studies published in the last few years confirm that IF can be a useful and safe therapeutical option for obesity and related disorders, no superiority to conventional caloric restriction diets have emerged. There are still several questions left answered. In this Review, we discuss some of the claims, unveiling myths, facts, and presumptions about several models of IF. The focus of this article is obesity, but there is a brief discussion of the potential benefits of IF on overall human health.

## INTRODUCTION

Intermittent fasting (IF) has received great interest from the general public, as an alternative to the traditional daily energy restriction model, for the treatment of obesity and related disorders, but also as an anti-aging method increasing longevity (1–3). Even though IF is a promising approach for some patients, there have been several claims of its benefits (mainly in social media) that are not evidence-based, at least in humans, leading to doubts among the society and sharp disagreements by its supporters and critics. As such, a critical appraisal of the literature is imperative to guide health professionals advising their patients, while not exaggerating its benefits, nor condemning its practice, yet also raising questions that may be answered by controlled trials in the future.

Recently, the medical community’s interest in this topic grew even further after the publication of a review article in the *New England Journal of Medicine* in late 2019 (1). That review article discusses, beyond obesity, several other potential targets for therapeutic fasting, such as the treatment of neurological disease, cancer, and cardiovascular disease. However, the article should be interpreted cautiously, as the authors clearly stated that the majority of the benefits were demonstrated in animal models, with much less data from humans. Moreover, in many of the cited articles, it is difficult to differentiate whether the effect of IF is a consequence of weight loss, caloric restriction, the fasting itself, or the interaction of these factors. This present Review aims to give a perspective of the evidence regarding intermittent fasting in humans, especially in the setting of metabolic diseases.

## INTERMITTENT FASTING: ONE NAME, SEVERAL PROTOCOLS

The term “Intermittent fasting” comprises several different forms of daily or weekly food intake patterns. Fasting is a common religious practice as well (4), but religious fasting will not be discussed in this article. Each IF protocol should be analyzed by itself regarding weight loss, metabolic and inflammatory profile, anti-oxidative effects, ketone bodies production, and adherence in the mid- and long-term. Circadian rhythms should also be taken into account, as similar feeding windows in different periods of the day could have a different cardiometabolic impact (5–7).

Every discussion considering different forms of fasting as having similar results is inevitably incomplete. Among the most studied IF protocols, three are summarized below and in Table 1 (3,4,8):

- Complete alternate-day fasting (ADF): involves alternating days containing no-energy food with eating *ad libitum* days;
- Modified alternate-day fasting (MADF): different variations in ADF. The most popular is “intermittent fasting 5:2”: allows five days per week of *ad libitum* feeding and makes absolute fasting or restricts the intake to no more than 25% of the daily necessity on the other two days (1,2). These two days can be consecutive or not consecutive;
- “Time-restricted feeding” (TRF): allows *ad libitum* intake during specific times (few hours) and fasting for a prolonged time during each day. Another variation is restricting intake throughout the night, following the circadian rhythm.

**Table 1:** Different patterns of IF

Type of Fast	Description
Complete alternate-day fasting (ADF)	Alternate fasting days with *ad libitum* eating days
Modified alternate-day fasting (MADF)	Variations in ADF – the most popular is the 5:2 diet: absolute fasting or severely restricting the caloric intake for two consecutive or non-consecutive days per week
Time-restricted feeding (TRF)	Daily intake is restricted to certain hours

## PROPOSED MECHANISMS MAINLY BASED ON ANIMAL MODELS

Most of the proposed direct mechanisms of intermittent fasting in diseases come from animal models, in which caloric restriction has been extensively associated with increased survival in many species. However, in most of these models, the animals tend to eat within a period of a few hours, generally in the active phase of their circadian day, and they fast during the rest of the 24-hour period. As such, it has been hypothesized that many of the benefits attributed to caloric restriction itself could be related to this long fasting period (1).

During fasting, there is a decrease in insulin levels and an increase in ketone bodies’ production, such as beta-hydroxybutyrate, a signaling metabolite that elicits several direct and indirect molecular responses (9). Those responses could reduce inflammation, oxidative stress, tumorigenesis, and aging (10). Among those signals, fasting and ketogenesis could increase AMPK and sirtuin gene expression, reduce mTor signaling (which is associated with cellular growth and cancer), increase mitochondrial biogenesis and autophagy and reduce sympathetic tone (1,9–11). Autophagy, in particular, is important for the removal of damaged cells and is associated with reduced aging and increased longevity in animals models (12). Indeed, many chronic diseases are associated with accumulation of dysfunctional cellular components and it is hypothesized that a shift from an anabolic post-prandial state to a catabolic state of reduced insulin is crucial for an effective autophagy to occur (1,12,13). Ketogenesis could also have an impact on epigenetic signaling by inhibiting histone deacetylases (9). Those mechanisms would positively have an impact on several diseases associated with inflammation, aging, and cellular growth, such as cancer, cardiovascular disease, type 2 diabetes, dementia, and auto-immune diseases (1,11). More immediate effects of ketone bodies’ production are enhanced lipolysis, reduced hunger, and improved mental and physical performance (including improved running endurance), which could influence the treatment of obesity (1,14). Interestingly, central infusion of ketone bodies potentiated leptin and insulin brain signaling (possibly affecting food intake) and directly improved hepatic insulin sensitivity, suggesting effects on insulin resistance, independent of weight loss (15). This direct effect of ketones on whole body insulin disposal has been shown in other animal models, as well (11,16). Ketones can also have a protein-sparing effect in obesity by inhibiting oxidation of branched chain amino acids, mainly alanine, in muscle (17). Nonetheless, studies performed decades ago observed that this protein sparing effect is dependent on the initial weight of participants, and individuals without obesity have increased loss of muscle mass during ketosis (17–19).

It has been proposed that living species should have mechanisms to adapt to long periods of food deprivation, with a clear metabolic switch from a fed to fasted state, occurring several times a week. In this context, the standard human pattern of three big meals a day, plus snacking, could be maladaptive, maintaining humans in a constant anabolic post-prandial state of increased insulin levels and no ketone bodies’ production (11).

A couple of published reviews have deeply discussed several of the above-mentioned molecular mechanisms (1–3,9,11), and we will focus this manuscript on human data from this point on.

## INTERMITTENT FASTING FOR TREATING OBESITY: IS IT SUPERIOR TO DAILY CALORIC RESTRICTION?

Many IF advocates claim in social media and books that this strategy increases energetic expenditure, leading to higher weight loss than daily caloric restriction (20). The theory defended by supporters is that part of its effect is secondary to a reduction in insulin levels (21), due to the carbohydrate-insulin model. This model states that hyperinsulinemia (elicited by excessive carbohydrate intake, mainly those with higher glycemic index) causes obesity by trapping metabolic fuels inside the adipose tissue. Consequently, eating would lead to a reduction in energy bioavailability to other tissues, including the brain, which, in turn, would interpret that the body is under an energy deficit, then adapting through increased hunger and reduced energy expenditure (21). Overall, this theory considers that the increased food intake and decreased energy expenditure are consequences, not causes, of obesity. Currently, although some authors still defend it (21,22), this theory has been widely discredited by experimental models in animals and humans (23–25). Also, isocaloric studies performed in metabolic chambers demonstrate that the differences in weight loss and energy expenditure among different macronutrients are clinically negligible (26–28). Although these studies did not evaluate fasting itself, but the differences between low-carb and low-fat diets, they reject the hypothesis that high insulin is, by itself, a major driver of weight gain in humans. Another study in a metabolic chamber evaluated time-restricted feeding (18 hours fasting) against 12 hours fasting in an isocaloric setting, and showed no difference in the weight loss between both groups (though there were differences in metabolic parameters, that are going to be discussed further ahead) (6). Other theoretical benefits on obesity treatment from intermittent fasting are the suppression of appetite (favoring caloric deficit), the improvement of physical disposition, and the improvement of metabolic parameters (2,4). As stated before, some of those mechanisms are related to increased ketogenesis. However, the increase in ketone bodies are different between different IF patterns, being higher in the fasting day of ADF, and probably mild in TRF models (11,29). Whether those increases could mimic the benefits of animal models of IF, in which larger ketone bodies production occurs, is uncertain. Interestingly, however, a study performing MADF (20% caloric intake on fasting days, alternate with *ad libitum* food intake every other day) in 10 individuals with asthma have shown, after 8 weeks, that even on *ad libitum* days, beta-hydroxybutyrate levels were significantly higher than in the baseline (29).

A systematic review searching for human trials published from January 1, 2000 to July 1, 2019 focusing on IF for weight loss, found only 18 small RCTs and 9 trials without active controls (30). In this study, the average reduction in body mass index was 4.3%, with weight losses ranging from 0.8% to 13%, and a high grade of heterogeneity. Still, it concluded that it was not possible to directly compare IF with other strategies of caloric restriction. Some of the most relevant studies will be discussed below.

Alternate-fasting (ADF) and modified alternate-day fasting regimens (MADF) are the most studied methods of IF to date. There are many studies with several different feeding and fasting protocols, such as the 5:2 and 3 or 4 days of energy restriction (Table 1). Short-term trials tend to show better results with IF (as adherence is higher), even though results are mixed (30). For instance, a study of 115 overweight women showed a higher fat mass loss in the group with MADF (2 days per week with 30% of the daily caloric needs) when compared to daily caloric restriction (75% of the daily caloric necessity); however, total weight, central adiposity, and hunger scores did not differ between the groups (31). Nonetheless, some studies show increased hunger in the first days of the 5:2 regimens and reduced hunger afterwards, but results are equivocal, and the overall evidence is not conclusive (4,32).

Trepanowski and cols. published a landmark study regarding obesity and MADF in 2017 (33). In this randomized controlled trial (RCT), 100 obese adults were assigned to 3 arms in a 6-month weight-loss phase, followed by a 6-month weight-maintenance period: alternate-day fasting (25% of energy needs on a fasting day, 125% of energy needs on the other day), calorie restriction (75% of energy needs every day), or no-intervention (control). The weight loss was higher in both the calorie restriction and IF group compared to control (mean of 6.8%) with no differences in the inflammatory and cardiovascular risk markers. However, a *post-hoc* analysis suggested that intermittent fasting provided increased insulin sensitivity among patients with previous insulin resistance (34). Interestingly, throughout the study, IF patients began to eat more calories on the restriction day and less on the “feast days,” approaching the standard diet, and the drop-out rate was higher in the IF group (38% vs. 29%). These findings clearly evidenced that a significant limitation of IF is adherence in the mid- and long-term (8,33).

Two other studies with different protocols and a one-year duration showed no difference between daily caloric restriction and IF, not only in weight, but also in markers such as glycated hemoglobin. Importantly, one of the studies was performed in patients with type 2 diabetes (35,36). In this way, medium- and long-term studies’ trials failed to show a superior benefit in weight loss from IF compared to traditional daily caloric restriction. However, this does not mean that it is a fad strategy. Recent studies are focusing on identifying good-responders (4,37,38). For instance, a *post-hoc* exploratory analysis of the trial performed by Trepanowski and cols., described above, assessed those “good-responder” patients, defined as those who lost more than 5% of their total body weight; regardless of the arm they were assigned. No differences were found regarding appetite and food intake behavior between the different diets groups in those “good responders” (37). However, some propose that “big eaters” would benefit less from MADF, as they can more easily overconsume on the days of regular feeding (4,38).

Fewer studies exist with complete ADF (total fasting on designated days). A recent one, in non-obese subjects, showed that four weeks of ADF led to a body weight reduction of 4.5% and an improvement in several cardiometabolic parameters, reduced visceral fat, increased polyunsaturated fatty acids levels (PUFAs), and reduced T3 levels (39). These results are similar to several models of caloric restriction and IF in animals (1,40,41). However, it lacks a control group with the same weight loss to evaluate weight-independent mechanisms of IF. Despite the controversy of the efficacy, it is essential to highlight that MADF and ADF are generally safe (1,2,4,39), with no reports of severe adverse effects. Metabolic adaptation after weight loss, an important obstacle for long-term weight maintenance, appears to be similar to DCR (42). IF does not increase the risk of an eating disorder (ED) (43), but it should not be indicated for patients with ED, as studies excluded these patients. There is a risk that proposing IF for these individuals can be mistakenly used to validate a chaotic eating pattern of restriction and indulgence.

“Time-restricted feeding” (TRF) trials are being published only in the last few years, yet it is probably the most popular type of IF in clinical practice. Defenders of this principle, base their arguments on the already mentioned animal models, in which over 16 hours of fasting per day improves metabolic markers and brings neuroprotection, which could be explained, at least partially, by an increase in ketone bodies (2). In humans, however, there is evidence that these same 16 hours just discretely increase ketone bodies, preventing any definitive conclusions on the overall benefit of this strategy without dedicated studies. (1,2,44,45).

Wilkinson and cols. performed a trial in which individuals who had a mean eating interval of 15.13 hours per day, reduced it to a mean of 10.78 h for 12 weeks (5). The individuals’ baseline status was used as the control, and the results showed a mean of 3.3 kg weight loss and a decrease in fat mass, LDL-cholesterol and non-HDL-cholesterol, blood pressure, and alterations of other metabolic factors. This was a single-arm study, and though mixed linear models suggested that metabolic improvements were not attributed to changes in weight, the lack of a control arm prevents a definite conclusion (5).

One pilot study involving 23 patients with obesity, compared to a historical control group, observed a 2.6% weight reduction with an 8-hour window from 10:00-18:00, without calorie counting, and a reduction in blood pressure (46). Recently, however, a larger RCT found more disappointing results (47). Lowe and cols. performed a randomized controlled trial in 116 overweight or individuals with obesity without imposing caloric restriction. One arm followed an 8-hour TRF (eating from 12:00-20:00) and the other received instructions for three structured meals a day. This exact feeding window was chosen to improve adherence, due to social and cultural reasons, allowing an early dinner. After 12 weeks, there was no difference in weight loss between the groups (-0.94 kg in the TRF versus -0.68 kg in the control arm), as well as no difference in general cardiometabolic parameters (glucose, insulin, lipids, and blood pressure). Fifty participants also performed a dual-energy-x-ray absorptiometry (DXA), and surprisingly, the TRF group had a higher decrease of appendicular lean mass compared to the control group. Indeed, 65% of the total weight loss in the TRF was lean mass, a result not expected, and a finding that deserves further analyses in future studies, which raises concerns on the applicability of this model in clinical practice. In the opposite direction, however, one study that evaluated 34 active men, performing resistance training randomized to a regular diet or TRF in a similar eight-hour window for eight weeks, showed fat mass (measured by DXA) decreased in TRF, without any decrease in muscle mass. An improvement of some inflammatory markers was also observed (48). Whether those differences on body composition observed relates to the different baseline characteristics of the studies participants (trained men versus individuals with overweight or obesity) remains to be determined.

Another recent study aimed to answer if a reduced feeding window could be more effective (49). In this study, patients with obesity were randomized into three groups for eight weeks: 6-hour feeding window (1300 h to 1900 h), 4-hour feeding window (1500 h to 1900 h), and control (maintenance of usual diet). Given the circadian rhythm (discussed below), feeding during the night was not allowed in neither of the active arms. Surprisingly, regardless of the regimen (4 h or 6 h of feeding), individuals with restricted feeding windows lost a mean of 3.2% of the baseline weight. The weight loss achieved was similar to what was observed in the above mentioned pilot study, from the same group, with an 08h feeding protocol (46). The effect is almost certainly related to reduced consumption of calories, due to the reduced feeding schedule: their caloric intake was decreased by approximately 550 kcal/day, but it is possible that this reduction in intake was overestimated, as a 500 kcal restriction usually leads to higher weight loss in an eight-week -period. As an additional benefit, insulin sensitivity improved and oxidative stress markers decreased in both intervention groups. In the discussion, the authors believe this effect could be independent from the weight-loss, although no definite conclusion can be made, as this trial did not include an arm for continuous caloric restriction without fasting (49).

Due to the low number of good-quality studies (that fortunately are increasing), we still have little evidence of a clear benefit of TRF compared to other protocols. The great advantage seems to restrict the consumption of calories during a period of the day, which, in the absence of super-compensation during the remaining hours, can reduce calories without the need to count them (4,46). Nonetheless, the decrease in muscle mass observed in the study by Lowe and cols. is disturbing (47). A question that arises is whether an equal feeding window performed early on in the day could have metabolic advantages and the role of physical activity in those results.

## TIME-RESTRICTED FEEDING AND CIRCADIAN RHYTHM

Biological control of external time is vital for all living species (2,7). In mammals, the circadian control is regulated by the suprachiasmatic nucleus in the hypothalamus – our “biological clock” (50). Circadian rhythms controlled by internal oscillators have an essential role in metabolic regulation (50,51). In evolution, each species tends to feed during the active period of their day (day or night, depending on the species). Several animal studies suggest that feeding during the rest period leads to an abnormal metabolic profile related to the ectopic deposition of fat, due to, among other reasons, inferior decreased lipogenic capacity during this period (52–54).

In humans, we know that individuals who have a later chronotype pattern have a worse metabolic profile and are exposed to an increased risk of weight gain, type 2 diabetes, and even cardiovascular outcomes. Still, many confounders prevent establishing a clear cause-effect (55–58). However, two RCTs performed one decade ago suggested that evening meals were associated with reduced fat mass, reduced HDL-cholesterol, increased LDL-cholesterol, increased blood pressure (in one of the trials), and increased fasting glucose (in the other one) (59,60). Recently, a study showed that after a weight-loss intervention protocol, late eaters had a lower weekly weight-loss rate compared to early eaters; however, the late eaters also had lower motivation and higher psychological barriers, suggesting that late eating could just be a marker of maladaptive eating, rather than it being biologically related to the poorer result (61).

Another study to help elucidate the role of circadian rhythm consisted of a supervised 5-week cross-over pilot study in men with prediabetes aimed to evaluate if TRF with a 6-h feeding window and dinner not after 1500 h had metabolic advantages over an isocaloric feeding pattern, with a 12 h feeding window and 12-h fasting. In this controlled study, the TRF period increased insulin sensitivity, reduced blood pressure, reduced oxidative stress, and decreased appetite (6). As it was an isocaloric study, it did not aim to evaluate food intake, and no weight differences were observed; thus, differences between control and intervention phases cannot be attributed to weight loss. However, as there was no similar group with the nighttime feeding period, it is impossible to know whether the effects were related to the 18 hours of fasting or the absence of nocturnal food intake. Simultaneously, as a pilot- and proof-of-concept study, it did not assessed the feasibility, so we cannot infer if this pattern is sustainable in a larger population for a longer period.

A recent RCT evaluated if late-night dinner (2200 h) could have a different metabolic impact than earlier dinner (1800 h) (62). Twenty healthy volunteers received the same meals on different days in a lab setting. Late dinner was associated with higher glucose, triglycerides peak delay, and reduced fatty acids oxidation, which could contribute to metabolic disturbances in the long term. Interestingly, however, the effects were more pronounced on habitual early sleepers, suggesting that individual social and biological differences in circadian rhythms can influence results. These differences have already been shown, regarding skipping breakfast, in which habitual “breakfast skippers” have very different responses than “breakfast eaters” (63,64).

Further, elucidation is needed for establishing causation for time-restricted feeding. Does regular breakfast skipping (or frequent nighttime eating) lead to metabolic adaptations? There is a possibility that the preference for eating or not eating breakfast or the time of having dinner is, at least partially, biologically determined, and a marker, instead of a cause, of a metabolic disorder.

## ADDITIONAL BENEFITS FROM INTERMITTENT FASTING

Many of the cited mechanisms of IF on animal models are related to the improvement of chronic conditions much beyond obesity itself.

Regarding type 2 diabetes (T2D), many of the proposed benefits of IF are similar to what has been previously discussed: weight loss and improved insulin sensitivity (1,30,65). A reduced number of meals can also be useful to avoid several glycemic excursions during the day. Some studies have shown that the reduction of the total number of meals can be a helpful strategy (65–67). In one study in women with T2D, eating two meals a day (in a maximum of a ten-hour interval) was superior to eating 6 smaller meals daily in weight loss and glycemic control (66). This is an important message, beyond IF itself. Historically, patients with T2D were advised to eat small amounts of food several times a day to avoid hypoglycemia. This recommendation, based on current knowledge, can be counterproductive (68). Clearly, in patients using exogenous insulin or sulfonylureas, dose adjustment will be necessary if IF is implemented, but overall, the risk of hypoglycemia is low (69).

The longer study performed evaluating IF for T2D to date (52 weeks), did not observe superiority of IF, regarding HbA1c or weight loss (36). In this context, IF regimens can be considered and individualized in patients with T2D, but similar to what was already discussed in obesity, it should be clear that it is an alternative to daily caloric restriction, rather than a superior intervention.

de Cabo and Mattson provided a very compelling and complete discussion of additional promising benefits of IF in cancer, aging, neuroprotection, and more (1). However, it is important to note that most of the available data discussed by de Cabo and Mattson come from caloric restriction experiments in animal models (1). Whilst in those models, caloric restriction is achieved through a TRF (as animals tend to eat in their active phase), it is not possible to conclude whether benefits are from fasting, caloric restriction, or both. Human studies are just emerging.

In the ADF study by Stekovic and cols., in a healthy, non-obese, middle-aged population, the age-associated inflammatory marker sICAM-1 was lower in the intervention arm (39). Aging is a complex biological process, and every attempt to prove that an intervention can slow it should be taken cautiously (70). RCTs designed to show anti-aging properties are tough to create, given the necessary long-term. Thus, metabolic and oxidative parameters are always used as surrogate markers, and as such, they are imperfect (71). Even with all these difficulties, we expect several studies to come about in the following years.

It is also important to reemphasize that we cannot directly extrapolate data from animal studies. If in rat models, high levels of ketone bodies are achieved after 16h fasting, in humans, it can take much longer, up to 48 hours, as already mentioned (11,44,45). In this context, since long-term fasting is harder to be performed, a group of researchers from California proposed a “fasting-mimicking diet” (FMD), which is patented and commercialized in the USA (72,73). FMD consists of plant-based, whole food diets, rich in essential fatty acids, vitamins, and minerals consumed for five consecutive days, which does not activate IGF1 and insulin pathways (74). As such, it would theoretically reproduce the scenario of fasting in the organism (72). The proposed benefits are reduced inflammation and aging, with a potential effect in several chronic non-communicable diseases. Although some studies showed improvement of inflammatory and oxidative stress markers, there is no evidence regarding significant clinical outcomes (73,74).

In conclusion, intermittent fasting has gained much attention as a popular weight-loss strategy over the last few years. Controlled studies demonstrated that IF (which can have very different protocols) is a safe alternative to daily caloric restriction, with similar weight loss results. Nonetheless, its effects on weight are directly related to caloric restriction and not the metabolic effects of fasting. More work is needed to establish if prolonged fasting can have a weight-independent impact on insulin sensitivity and other metabolic and oxidative markers in humans, as shown in animals. A comprehensive summary of the main unanswered questions can be found in Table 2.

**Table 2:** Unanswered questions and rationale for them

Unanswered questions about IF protocols	Rationale for the question
Does time-restricted feeding during the morning, afternoon, or evening have different metabolic effects?	Circadian rhythm is biologically regulated, and many animals’ studies demonstrate metabolic disarrangements when food intake occurs in the rest phase of the species.
Are there weight-loss independent effects of intermittent fasting on cardiovascular risk markers and insulin resistance?	Prolonged fasting has a unique metabolic profile in animals, and some studies in humans suggest that insulin sensitivity can improve, even in the absence of weight loss. Others suggest a beneficial effect in those with insulin resistance.
Can fasting-mimicking diets reduce aging or chronic diseases?	The rationale for the development of a fast-mimicking diet is to provide these benefits. Nowadays, we only have evidence of improved surrogate markers.
Is there an intermittent fasting protocol associated with higher long-term adherence?	Fewer studies compared different intermittent fasting protocols head-to-head. Long term adherence is important for sustained health benefits.

Results from trials are heterogeneous, and due to that, a personalized approach is necessary. The search for clinical markers able to predict responses is appealing. As most trials are small, many discussed results should be interpreted cautiously. Lack of statistical power to show differences in weight loss and substitutive markers is one crucial factor that should be considered when evaluating IF RCTs.

While there is no definitive conclusion, IF clearly has some advantages and disadvantages. A summary of the popular claims pro and con to IF, as well as the current state of the evidence can be found in Table 3. Against IF, long-term trials suggest lower adherence in the long-term compared to daily caloric restriction (or an increase in caloric intake on fasting days and a decrease in feeding days that resembles a routine caloric restriction protocol). Favoring MADF and ADF is that it can be easier to follow, since it generally is not necessary to count calories, or at least permits regular eating on the majority of the days. Regarding TRF, the imposed restriction on timing, rather than foods, can also be easier to follow for some. Different IF protocols can have direct effects on insulin sensitivity in insulin-resistant individuals (although data are still equivocal), and restricting food at night could also have a positive impact on the metabolic profile. Nevertheless, “inversed” TRF, with food restriction in the morning, is less studied and theoretically goes against our circadian biology.

**Table 3:** Popular claims (pro and agains) to IF regimens

Popular positive claims	Current status of evidence
Increased weight loss compared to isocaloric diets (lower insulin and ketogenesis would increase energy expenditure).	There is no evidence that IF leads to higher energy expenditure: isocaloric studies performed in metabolic chambers show similar weight loss in diets with different proportions of macronutrients (27); RCTs show the same weight loss for daily caloric restriction and intermittent fasting, while maintaining similar weekly caloric intake (33,36,37) and no difference in adaptive thermogenesis (42).
Reduced hunger compared to traditional daily caloric restriction.	Conflicting results in humans (4). Studies with a proper control group did not show an overall difference in hunger ratings, though individual preferences exist (30,32,38).
Improvement in insulin resistance, independent of weight loss.	Data derived from animal models (2,11,16). In humans, conflicting results, but many studies without a proper control group. Overall, there is no clear evidence of improvement in IR, independent of weight loss (4). However, *a post hoc* analysis suggests benefits in those who had previous insulin resistance (34). Timing of food intake could also have a role (6).
Improvement in glucose control in type 2 diabetes.	RCTs show similar reductions in weight and HbA1c (36,65). However, there is evidence that increased meal frequency is associated with poorer glycemic control (66,67).
Anti-aging and anti-inflammatory effects.	Many animal models demonstrate increased longevity and reduction in chronic diseases with caloric restriction and IF, but data in humans are lacking (1,11). Many benefits are alleged to be associated with ketogenesis, but it is unclear whether the level of ketone bodies achieved in popular IF patterns would be enough to elicit the same responses observed in rodents (11). Fasting-mimicking diets have been developed to promote these benefits and have shown a reduction in inflammatory and oxidative stress markers, but not clinical outcomes to date (72–74).
Popular negative claims	Current status of evidence
IF would lead to overeating on feast days and could trigger eating disorders.	IF does not lead to overeating or bingeing on feast days and does not trigger eating disorders (43). Nonetheless, patients with previous eating disorders were excluded from main trials, and IF is not recommended for this population.
IF could reduce lean mass.	Conflicting results. Several studies suggest positive effects on lean mass, including one in trained men (48). However, a recent randomized trial with TRF observed a higher appendicular lean mass loss after the intervention (47). More data are needed.
IF is not sustainable and leads to higher attrition rates.	Conflicting results, but IF has a slightly higher attrition rate in larger trials (4,33). One key trial has shown that with MADF, there was a trend of increasing calories on fasting days and decreasing on feasting days, resembling the overall daily calorie restriction pattern (33).
IF leads to side effects, such as nutrient deficiency and lethargy.	Trials with different IF patterns show it is generally safe and well-tolerated, with no increase in overall side effects (4).

Benefits on aging, inflammation, and neuroprotection in humans are still scarce. Fasting mimicking-diets are being studied with interesting results, and we can expect further elucidative research in the area.
